# Bilateral Pelvic Ectopic Kidneys With Bicornuate Uterus: A Rare Case of Congenital Association Complicated by Urolithiasis and Pregnancy

**DOI:** 10.7759/cureus.110583

**Published:** 2026-06-10

**Authors:** Abdulaziz Al Shaibani, Hasan F Buali, Noora Aljeeran, Khalifa Albenjasim, Mohamed Rafie

**Affiliations:** 1 Urology, Bahrain Defence Force Hospital, Riffa, BHR; 2 Urology, King Hamad University Hospital, Muharraq, BHR

**Keywords:** bicornuate uterus, cakut, müllerian anomaly, pelvic kidney, renal ectopia, urolithiasis

## Abstract

Bilateral pelvic ectopic kidneys represent an exceptionally rare congenital anomaly. Their co-occurrence with Müllerian anomalies reflects shared embryological disruption of the metanephric and paramesonephric systems and carries significant clinical implications, particularly during pregnancy. A 37-year-old woman with no previous urological history presented with left flank pain. Renal ultrasound demonstrated bilateral pelvic ectopic kidneys and a 5 mm non-obstructing left renal calculus. Oral analgesia and increased fluid intake were advised. Repeat ultrasound six months later confirmed spontaneous passage of the calculus with no residual stone burden. The patient was concurrently under gynecological follow-up for a bicornuate uterus. A non-contrast pelvic magnetic resonance imaging (MRI), performed by the gynecology department, to characterize the uterine anomaly and deferred to a non-contrast protocol due to concurrent pregnancy, confirmed bilateral ectopic kidneys situated in the presacral and rectovaginal pouch of Douglas with aberrant vascular supply arising near the common iliac artery bifurcation. Renal function tests and urinalysis remained within normal limits throughout the follow-up period, including during pregnancy. The patient delivered at 37+3 weeks of gestation by elective cesarean section without maternal or urological complications and was maintained under urological surveillance post-partum, with computed tomography urography planned for full anatomical delineation. This case illustrates a rare embryologically linked combination of bilateral pelvic ectopic kidneys and a bicornuate uterus. Awareness of this association may aid multidisciplinary management across urology, gynecology, and obstetrics. Consideration should be given to evaluation for co-existing Müllerian anomalies when congenital renal anomalies are identified, and vice versa.

## Introduction

Renal ectopia results from failure of the metanephros to complete its normal cephalad ascent during the fourth to eighth weeks of embryogenesis [1]. The ectopic kidney occurs in approximately 1 in 3,000 individuals, with bilateral pelvic ectopia representing a far rarer subset, reported in fewer than 10% of all renal ectopia cases [2]. Pelvic ectopic kidneys are frequently associated with anomalous vascular supply, malrotation, and upper urinary tract complications, including ureteropelvic junction obstruction and urolithiasis [2,3].

Congenital anomalies of the kidney and urinary tract (CAKUT) and Müllerian anomalies frequently co-occur, likely reflecting disruption of the closely related embryological development of the mesonephric and paramesonephric systems [4]. The Müllerian duct, which gives rise to the fallopian tubes, uterus, cervix, and upper vagina, forms alongside the Wolffian duct, which gives rise to the ureteric bud and drives metanephric development [4]. Disruption before the eighth gestational week can therefore simultaneously impair renal ascent and Müllerian fusion [4,5]. The prevalence of Müllerian anomalies in women with a diagnosed renal anomaly has been reported at approximately 29%, though this is considered an underestimate given incomplete screening in many patients [4]. Identification of a renal anomaly should therefore prompt consideration of evaluation for concurrent Müllerian anomalies, and vice versa.

The ectopic renal position may complicate the interpretation of pelvic imaging and introduce significant risk during obstetric and gynecological procedures [6]. The gravid pelvic uterus in a patient with bilateral pelvic kidneys creates an anatomically constrained environment that may limit imaging quality during pregnancy [7]. We report a case of bilateral pelvic ectopic kidneys co-existing with a bicornuate uterus, presenting with left renal colic and managed across a multidisciplinary setting. To our knowledge, this specific combination, complicated by concurrent urolithiasis and pregnancy, has not been previously described as a combined clinical entity in the literature. This is a single-patient case report and does not constitute research involving human subjects requiring formal IRB approval.

## Case presentation

A 37-year-old primigravida with no previous urological or medical history presented to the urology outpatient clinic with a single episode of left flank pain. No hematuria or urinary symptoms, including dysuria, frequency, or urgency, were present. Physical examination was unremarkable.

Renal and bladder ultrasound demonstrated bilateral ectopic kidneys located within the pelvis, with preserved corticomedullary differentiation, normal renal parenchymal thickness, and normal echogenicity. A 5 mm non-obstructing calculus was identified within the left kidney. No back-pressure changes were identified bilaterally (Figures 1-2). The urinary bladder demonstrated normal wall thickness with no lesions or calculi.

**Figure 1:**
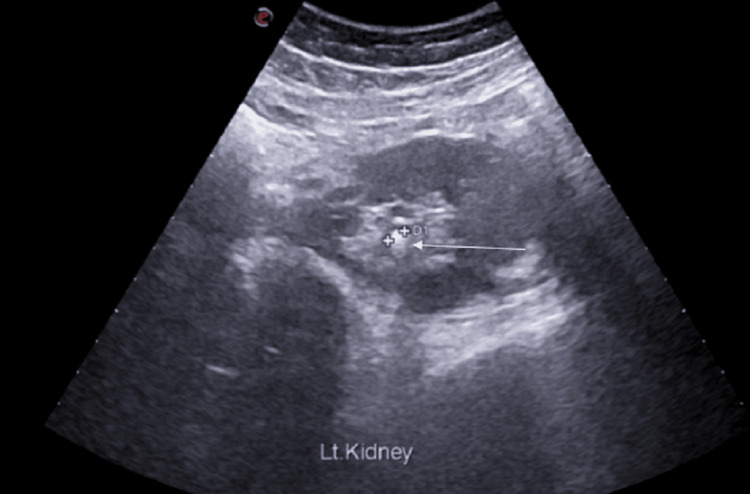
Pelvic ultrasound demonstrating a 5 mm echogenic focus with posterior acoustic shadowing within the left ectopic pelvic kidney. The arrow indicates the 5 mm left renal calculus.

**Figure 2:**
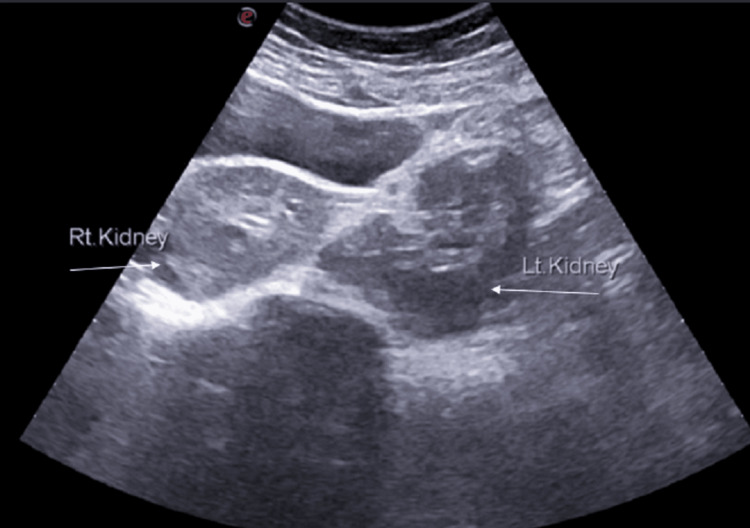
Pelvic ultrasound demonstrating bilateral ectopic kidneys located within the pelvis with preserved corticomedullary differentiation and no evidence of back-pressure changes bilaterally. Arrows indicate bilateral ectopic pelvic kidneys.

Conservative management was elected given the absence of obstruction, infection, and renal impairment. Oral analgesia and increased fluid intake were advised in accordance with the current guidelines [3]. Renal function tests were within normal limits, and urinalysis was negative for microscopic hematuria, leukocyturia, and nitrites at presentation and remained stable throughout the follow-up period.

Approximately six months following the initial presentation, the patient was confirmed pregnant. She was under concurrent gynecological follow-up for a known bicornuate uterus, for which a cervical cerclage was placed at 13 weeks of gestation, given the recognized risk of cervical incompetence associated with this uterine anomaly.

At repeat ultrasound performed at 37 weeks of gestation, bilateral pelvic ectopic kidneys were again demonstrated with preserved corticomedullary differentiation and no back-pressure changes bilaterally. No renal calculus was identified on this study, consistent with spontaneous passage of the previously documented left renal stone; passage was inferred radiologically by the absence of the calculus rather than by stone retrieval. Technical limitations were noted due to the gravid uterus.

A non-contrast pelvic MRI was performed by the gynecology team at 37 weeks of gestation to characterize the uterine anomaly and delineate renal anatomy before planned delivery, with gadolinium-based contrast withheld in accordance with the standard radiological practice during pregnancy [7]. MRI confirmed bilateral ectopic kidneys situated deeply within the pelvis in the presacral and rectovaginal pouch of Douglas, positioned posterior to the lower uterine corpus and uterine cervix (Figures 3-4). Both renal arteries arose from single trunks originating near the common iliac artery bifurcation, with preserved flow voids. Venous drainage entered the common iliac veins bilaterally. The ureters were not traceable on the non-contrast study. The gravid uterus occupied the central pelvis, producing technical limitations in the assessment of surrounding structures. No significant back-pressure changes were identified in either kidney. The bicornuate uterus was confirmed as a concurrent finding. Early degenerative changes were noted at the bilateral sacroiliac joints as incidental findings; whether these reflect chronic mechanical stress from the anatomically congested presacral space, occupied by bilateral ectopic kidneys and a bicornuate uterus, cannot be excluded.

**Figure 3:**
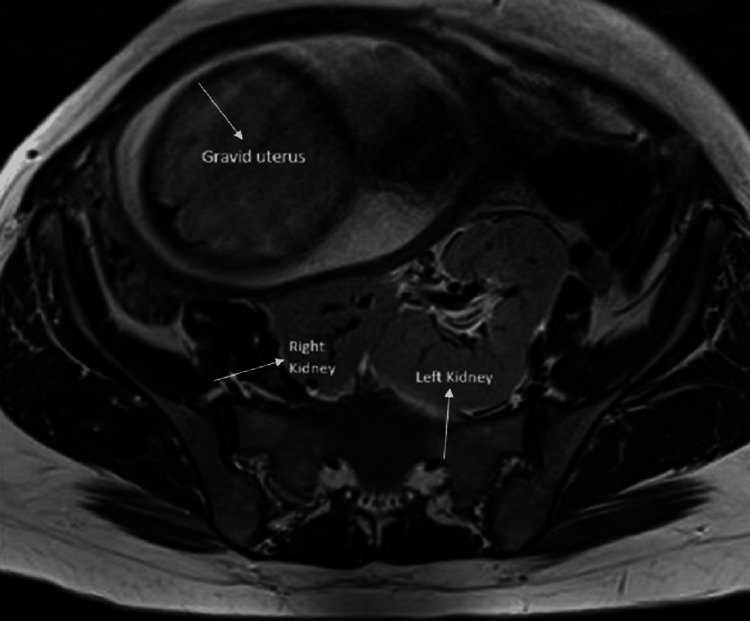
Axial non-contrast T2-weighted pelvic MRI demonstrating bilateral ectopic pelvic kidneys situated within the pelvis, positioned posterior to the gravid bicornuate uterus. Arrows indicate bilateral ectopic kidneys and a gravid bicornuate uterus. MRI: magnetic resonance imaging

**Figure 4:**
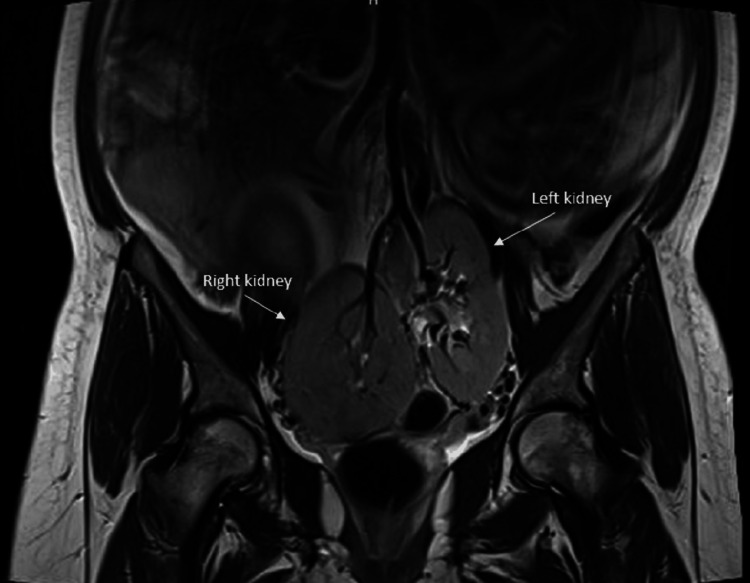
Coronal non-contrast T2-weighted pelvic MRI demonstrating bilateral ectopic pelvic kidneys (white arrows) situated in the presacral and rectovaginal pouch of Douglas, posterior to the gravid bicornuate uterus MRI: magnetic resonance imaging

CT urography was deferred, given concurrent pregnancy, in keeping with the current guideline recommendations [3]. The fetus was noted to be in an oblique lie throughout the third trimester, a malpresentation directly attributable to the bilateral pelvic ectopic kidneys occupying the presacral space and displacing the lower uterine segment, thereby preventing normal cephalic engagement. Given this malpresentation and the maternal pelvic renal anatomy, elective cesarean section was planned. The case was discussed at a multidisciplinary level between the obstetric and urology teams. A consultant urologist was present at the time of surgery, given the unknown ureteric course on non-contrast imaging and the proximity of both ectopic kidneys to the operative field. The patient delivered at 37+3 weeks of gestation by elective lower segment cesarean section. A live male infant was delivered weighing 2,907 kg with no maternal or urological complications. She presented for post-partum urological review 10 months following delivery, remaining asymptomatic with no recurrent flank pain, hematuria, or urinary tract infections. Total follow-up from the initial presentation to the time of this report spans approximately 25 months. Renal function tests and urinalysis remained within normal limits throughout. CT urography has been requested as part of ongoing post-partum surveillance to fully delineate ureteric anatomy, exclude residual urolithiasis, and characterize the vascular supply of both ectopic kidneys before any future pelvic intervention; results are pending at the time of submission.

## Discussion

This case demonstrates the co-occurrence of bilateral pelvic ectopic kidneys and a bicornuate uterus, a combination with distinct clinical implications across urology, gynecology, and obstetrics.

The embryological basis for this association lies in the shared developmental pathway of the renal and genital systems. The Müllerian duct develops alongside the Wolffian duct, which gives rise to the ureteric bud and drives metanephric differentiation and renal ascent. Disruption of this process can simultaneously impair both renal ascent and Müllerian fusion [4,5]. Genes including *HNF1B*,* GATA3,* and *WNT4* regulate both renal and genital tract development, and mutations in these genes have been associated with combinations of renal anomalies and Müllerian malformations, including bicornuate uterus and uterovaginal aplasia [4]. The co-occurrence of bilateral renal ectopia and bicornuate uterus in this patient may warrant consideration of genetic counseling given the potential for heritable disruption of urogenital development, though genetic testing was not performed in this case [4,5].

The kidneys in this case were positioned in the presacral and rectovaginal pouch of Douglas, posterior to the uterine corpus and cervix. This location has direct implications for any future pelvic surgical intervention. Kedia et al. described laparoscopic radical hysterectomy in the presence of a unilateral pelvic ectopic kidney and emphasized that thorough preoperative characterization of renal position, ureteric course, and vascular anatomy is essential [6]. In bilateral pelvic ectopia, where both kidneys and their anomalous vasculature occupy the operative field, this requirement is even more critical. Beyond Müllerian anomalies, patients with bilateral pelvic ectopic kidneys should also be evaluated for associated urological anomalies, including vesicoureteral reflux, ureteropelvic junction obstruction, and ureteric duplication, as these co-occur in a significant proportion of cases [2]. Aberrant renal arteries arising near the common iliac bifurcation, as seen in this case, are a recognized feature of pelvic ectopic kidneys and must be mapped before any planned pelvic surgery [2].

The 5 mm left renal calculus was managed conservatively with successful spontaneous passage, inferred radiologically on repeat ultrasound. Current European Association of Urology (EAU) guidelines report a spontaneous passage rate of approximately 75% for stones under 5 mm, supporting the expectant approach adopted here [3]. Urinary stasis secondary to malrotation and anomalous ureteric drainage nonetheless predisposes ectopic kidneys to recurrent stone formation, and long-term stone surveillance is warranted [2,3]. CT urography was appropriately deferred due to concurrent pregnancy; EAU guidelines recommend ultrasound as first-line imaging in pregnant patients with suspected urolithiasis, with MRI as the second-line option and low-dose CT reserved as a last resort [3]. Non-contrast MRI provided adequate characterization of the kidneys and uterus in this case, but was limited in tracing the ureters, a recognized constraint of this modality. Conti-Ramsden et al. demonstrated that pelvicalyceal diameters on MRI in pregnancy are systematically higher than those on ultrasound, with upper reference limits of 17 mm on the left and 25 mm on the right, which must be considered when interpreting pelvicalyceal appearances in pregnant patients with ectopic kidneys [7].

The gravid uterus expanding into a pelvis already occupied by bilateral ectopic kidneys raises a theoretical risk of extrinsic ureteric compression. In the normal gravid pelvis, physiological hydronephrosis occurs due to mechanical and hormonal effects [7]. In bilateral pelvic ectopia with anomalous shorter ureteric courses, this risk may theoretically be heightened; however, no ureteric obstruction was demonstrated in this patient, and the pregnancy was completed without urological complication, with consistently stable renal function. A particularly significant obstetric consequence in this case was persistent fetal oblique lie throughout the third trimester, directly attributable to the bilateral pelvic ectopic kidneys physically preventing normal cephalic engagement. This malpresentation formed a co-indication for elective cesarean section, and a consultant urologist was present at the time of surgery, given the proximity of both kidneys to the operative field and the unknown ureteric course on non-contrast imaging.

The co-existing bicornuate uterus adds further clinical relevance. Its identification alongside bilateral pelvic ectopic kidneys should prompt multidisciplinary review involving urology, obstetrics, and clinical genetics. Reinoso Calle reported a case of Mayer-Rokitansky-Küster-Hauser syndrome type II incorporating renal ectopia and multisystemic anomalies, illustrating that the spectrum of co-occurring urogenital malformations is broad and crosses multiple specialties [5]. The present case reinforces the principle that identification of one anomaly in this embryological spectrum should prompt consideration of evaluation for the other [4,5].

Four management principles emerge from this case. First, identification of bilateral pelvic ectopic kidneys should prompt consideration of evaluation for concurrent Müllerian anomalies and associated urological anomalies, including vesicoureteral reflux, ureteropelvic junction obstruction, and aberrant vascular anatomy, given their shared embryological basis [2,4,5,6]. Second, urolithiasis in pelvic ectopic kidneys should be managed in line with current guidelines, with imaging modality adapted to the clinical context, including pregnancy [3]. CT urography should follow pregnancy-related deferral as part of post-partum surveillance, as planned in this case. Third, pregnancy in the setting of bilateral pelvic ectopic kidneys warrants coordinated multidisciplinary surveillance; fetal malpresentation from mechanical displacement of the presenting part should be anticipated, and a urologist should be available at the time of any pelvic surgical intervention. Fourth, the theoretical risk of ureteric compression in pregnancy should be acknowledged but must be distinguished from directly observed findings; in this case, no obstruction occurred, and renal function remained stable throughout.

## Conclusions

Bilateral pelvic ectopic kidneys co-existing with a bicornuate uterus represent a rare congenital combination reflecting shared disruption of urogenital embryogenesis. This case demonstrates that such patients may present initially with urological symptoms and that concurrent Müllerian anomalies should be considered upon identification of renal ectopia. Pregnancy in this anatomical context introduces diagnostic and surveillance challenges requiring coordinated multidisciplinary management. Preoperative imaging to map renal position, ureteric course, and vascular anatomy is essential before any future pelvic intervention. In this case, a cesarean section was performed in the presence of bilateral pelvic ectopic kidneys, highlighting the real rather than theoretical surgical risk this anomaly presents. Long-term urological surveillance is warranted given the recognized association of pelvic ectopic kidneys with upper urinary tract complications. This case highlights important clinical considerations regarding the coexistence of bilateral pelvic ectopic kidneys and a bicornuate uterus; however, further case reports and larger studies are required to better define optimal surveillance strategies, genetic associations, and pregnancy-related outcomes in this rare congenital combination.
